# Identifying individuals with virologic failure after initiating effective antiretroviral therapy: The surprising value of mean corpuscular hemoglobin in a cross-sectional study

**DOI:** 10.1186/1742-6405-7-25

**Published:** 2010-07-23

**Authors:** Bryan Lau, Geetanjali Chander, Stephen J Gange, Richard D Moore

**Affiliations:** 1 Department of Epidemiology, Johns Hopkins Bloomberg School of Public Health, 615 N. Wolfe Street, Baltimore, Maryland 21205, USA; 2 Department of Medicine, Johns Hopkins University School of Medicine, 1830 E. Monument Street, Baltimore, Maryland 21287, USA

## Abstract

**Objective:**

Recent studies have shown that the current guidelines suggesting immunologic monitoring to determine response to highly active antiretroviral therapy (HAART) are inadequate. We assessed whether routinely collected clinical markers could improve prediction of concurrent HIV RNA levels.

**Methods:**

We included individuals followed within the Johns Hopkins HIV Clinical Cohort who initiated antiretroviral therapy and had concurrent HIV RNA and biomarker measurements ≥4 months after HAART. A two tiered approach to determine whether clinical markers could improve prediction included: 1) identification of predictors of HIV RNA levels >500 copies/ml and 2) construction and validation of a prediction model.

**Results:**

Three markers (mean corpuscular hemoglobin [MCH], CD4, and change in percent CD4 from pre-HAART levels) in addition to the change in MCH from pre-HAART levels contained the most predictive information for identifying an HIV RNA >500 copies/ml. However, MCH and change in MCH were the two most predictive followed by CD4 and change in percent CD4. The logistic prediction model in the validation data had an area under the receiver operating characteristic curve of 0.85, and a sensitivity and specificity of 0.74 (95% CI: 0.69-0.79) and 0.89 (95% CI: 0.86-0.91), respectively.

**Conclusions:**

Immunologic criteria have been shown to be a poor guideline for identifying individuals with high HIV RNA levels. MCH and change in MCH were the strongest predictors of HIV RNA levels >500. When combined with CD4 and percent CD4 as covariates in a model, a high level of discrimination between those with and without HIV RNA levels >500 was obtained. These data suggest an unexplored relationship between HIV RNA and MCH.

## Introduction

Current World Health Organization guidelines recommend using CD4 counts to monitor treatment response to highly active antiretroviral therapy (HAART) in regions where HIV viral load testing is unavailable [1]. However, recent reports suggest that monitoring CD4 counts does not accurately classify individuals who have not successfully suppressed HIV RNA levels [2-4]. One study, from Uganda, examined whether CD4 counts and CD4 percentages could be used to classify individuals as above or below four thresholds of HIV RNA (50, 500, 1000, and 5000) and at three time points (6, 12, and 18 months) after the initiation of treatment [3]. Various classification schemes based upon CD4 counts (e.g. an increase in CD4 count from 0 to 6 months) or CD4 percentage provided a sensitivity range of only 0.04-0.62 for detecting individuals with HIV RNA above 500 [3]. We examined whether other clinical markers that are routinely assessed within the Johns Hopkins HIV Clinical Cohort (JHHCC) could provide better classification of individuals who do not have suppressed HIV RNA levels using a novel approach.

## Methods

The JHHCC was established to prospectively quantify the processes and outcomes of care for HIV-infected individuals seen in clinical practice in the Baltimore metropolitan area [5]. All patients give informed consent and the JHHCC is conducted in accordance with the ethical standards of the Johns Hopkins Institutional Review Board and with the Helsinki Declaration of 1975. Subjects included in this analysis were individuals who initiated HAART after January 1, 2000 and had an HIV RNA measurement at least 4 months after initiation. Each individual also had to have at least one of the biological markers (listed below) measured within 60 days before or 30 days after the time of HIV RNA measurement. Only a single record of HIV RNA (the first measurement occurring at least 4 months after HAART initiation) and clinical markers for each individual was included in the analyses. All individuals were still on treatment at the time of their HIV RNA measurement.

We utilized a random forest approach to evaluate the ability of routinely collected clinical markers to classify individuals as greater or less than 500 HIV RNA copies/ml. Random-forests are an algorithmic, non-parametric approach to identify prognostic variables and are robust to over fitting the data [6,7]. These methods are an extension of classification and regression trees (CART) which by introducing randomness in variable selection and have been shown to have lower error and better classification rates [6,8]. Briefly, individual classification trees were generated from random bootstrap samples from the data set. Each node of the tree (or branch point) was created by selecting a random subset of candidate classification variables. As with standard CART methods, nodes were split by variables that optimize a splitting criteria and each tree is grown to full size. Because each classification tree was developed from a bootstrap sample of the study population, a subset of the study population remained unused for that tree; this subset was used to validate the tree and estimate the classification error. Ultimately, the random forest approach provides a measure of each variable's importance by examining (in the validation subset) the increase in error rate when the variable is ignored [6,8]. This consists of running the data from the subset of individuals not chosen in the bootstrap sample through the tree while permuting each covariate in turn. Thus each covariate has a set of error rates (obtained from each tree) for when the specific covariate has and has not been permuted. The change in the error rate is summarized over all trees in the random forest and divided by the standard error to provide a standardized change in error rate. If a variable did not truly have prognostic importance then the change in error rate should be distributed around 0 and normally distributed.

The random-forest approach was used to search for prognostic variables among the following measures: absolute CD4, percent CD4, serum albumin, alanine aminotransferase, aspartate aminotransferase, creatinine, hemoglobin, total lymphocyte, eosinophil, and neutrophil counts, potassium, calcium, chloride, CD3 counts, red blood cell count, mean corpuscular hemoglobin (MCH), mean corpuscular hemoglobin concentration (MCHC), mean corpuscular volume (MCV), packed cell volume, platelet count, alkaline phosphatase, CO_2 _, direct billirubin, and HAART regimen (protease inhibitor [PI], non-nucleoside [NNRTI], triple nucleoside [NRTI], and dual regimen containing both PI and NNRTI based regimens). The measured value up to 1 year preceding HAART initiation, and the corresponding difference between post-HAART and pre-HAART values, were included (e.g. change in MCH = [post-HAART MCH] - [pre-HAART MCH]). Individuals who were missing marker measurements had values imputed using the approach for imputation in random-forests as outlined by Brieman [9] (R Foundation for Statistical Computing, Vienna, Austria: http://www.R-project.org**)**. Imputation in random forests maintains accuracy when up to 80% of the data are missing [9].

Initially the random forest included all covariates to determine an overall error rate and order of the variable importance. Utilizing this information, we constructed another random forest limiting the covariates to the 12 most important variables. Subsequently we continued to prune covariates from the random forest by eliminating the least predictive variables until we reached a random forest consisting of variables which had a variable importance above 1.96 as a cutoff since a non-prognostic variable should be normally distributed.

While, the random-forest approach provides an excellent method for identification of important, prognostic variables, it unfortunately does not produce a familiar regression equation that can be easily disseminated through printed material. Furthermore, the random-forest may contain thousands of trees and therefore cannot be easily included in a figure. Thus, we utilized the random-forest results to identify variables that had the most predictive capability based upon the variable importance measure. We then used these variables to construct a logistic model with HIV RNA above 500 cps/ml as the outcome and the concurrent markers (or change from pre-HAART levels) were included as covariates. Because of the missing data, we re-imputed the missing data 20 times to account for the variability in the imputation process and summarized the results of the logistic model for multiple imputation [10]. To optimally assess the classification error of both the random-forest and logistic model, we reserved one-half of the study population as a cross-validation set. The models calibration was examined by splitting the predicted probabilities from the logistic model into 8 quantiles (each consisting of 98 indviduals) and assessing the observed probability of having an HIV RNA above 500 copies/ml as compared to the mean predicted probability in each quantile group [11].

To determine how well the models were able to discriminate between those who did and did not have an HIV RNA above 500 copies/ml, we relied primarily on the receiver operating characteristic (ROC) curve and the area under the receiver operating characteristic (AUROC) curve. An ROC curve is the relationship between sensitivity and specificity when different cutoff of a distribution is utilized. The AUROC provides the probability that one can discriminate between two individuals (one randomly chosen from those who are above 500 copies/ml and one randomly chosen from those that are below 500 copies/ml) which individual is above 500 copies/ml [12].

## Results

The study population was comprised of 1,568 individuals; 784 were used for the random-forest analysis and 784 for the validation set. Study population characteristics are shown in Table 1. The median (interquartile range, IQR) time the HIV RNA was measured after HAART initiation was 0.48 (IQR: 0.39-0.65) years. The majority of individuals had an HIV RNA level below 500 copies/ml (1017 [65%]). The median CD4 count just prior to HAART initiation was 190 (IQR: 66-315) cells/mm^3 ^and 278 (IQR: 143-426) at the time of HIV RNA measurement. Most were on a PI-based regimen (47%) followed by a NNRTI-based regimen (38%) with the rest on either a dual PI and NNRTI or triple NRTI-based regimen (15%). A total of 696 (59%) were on a regimen containing a thymidine analogue (49% containing zidovudine and 33% containing stavudine).

**Table 1:** Study population characteristics

	Training Data (n = 784)	Validation Data (n = 784)
Median Age (IQR)	42.4 (36.7, 47.6)	41.8 (36.2, 48.3)
Male Sex - N (%)	500 (64)	522 (67)
Race - N (%)		
African-American	588 (75)	589 (75)
White	169 (22)	174 (22)
Other	27 (3)	21 (3)
HIV Risk Behaviors - N (%)*		
MSM	203 (26)	203 (26)
IDU	288 (37)	290 (37)
Heterosexual	412 (53)	399 (51)
Median RNA (IQR) copies/ml**	155 (50, 6147)	145 (50, 7071)
Median CD4 (IQR) cells/ul**	273 (149, 441)	279 (133, 418)
	[N = 701 (89%)]†	[N = 695 (89%)]†
Median Change in Percent CD4 (IQR)***	2.9 (0.0, 6.7)	3.0 (-0.4, 7.3)
	[N = 655 (84%)]†	[N = 635 (81%)]†
Median MCH (IQR) (pg/cell)**	33.0 (30.2, 36.6)	32.8 (29.9, 36.2)
	[N = 413 (53%)]†	[N = 410 (52%)]†
Median Change in MCH (IQR)***	1.7 (-0.2, 4.3)	1.8 (-0.3, 5.2)
	[N = 364 (46%)]†	[N = 360 (46%)]†

*HIV risk behaviors are reported behaviors at enrollment into the cohort and are not mutually exclusive

** At time of first HIV RNA measurement at least 4 months after initiation of effective therapy

*** Change is the change from pre-HAART levels to marker measurement concurrent with HIV RNA measurement occurring at least 4 months after initiation of treatment.

† The number and percent in brackets correspond to the number of individuals that were not missing these data.

Utilizing the variables listed above, the random-forest method was able to correctly classify 659/784 individuals: 473/509 individuals with HIV RNA < 500 copies/ml, for a specificity of 0.93 [95% confidence interval, CI: 0.90-0.95] and 186/275 individuals ≥ 500 copies/ml, for a sensitivity of 0.68 [95% CI: 0.62-0.73]. The most important variable when all variables were included was the MCH levels followed by change in MCH from pre-HAART levels. Using the variable importance as a guide, a new random-forest was grown eliminating the least important variables from the model. The final random-forest included with four different markers: MCH (both current and change from pre-HAART level), current CD4 count, change in percent CD4, and MCHC (both current and change from pre-HAART level). This final, reduced random-forest was able to correctly classify 643/784 individuals for an overall error rate of 18% (specificity: 459/409 = 0.90 [95% CI: 0.87-0.93]; sensitivity: 184/275 = 0.67 [95% CI: 0.61-0.72]).

**Figure 1:**
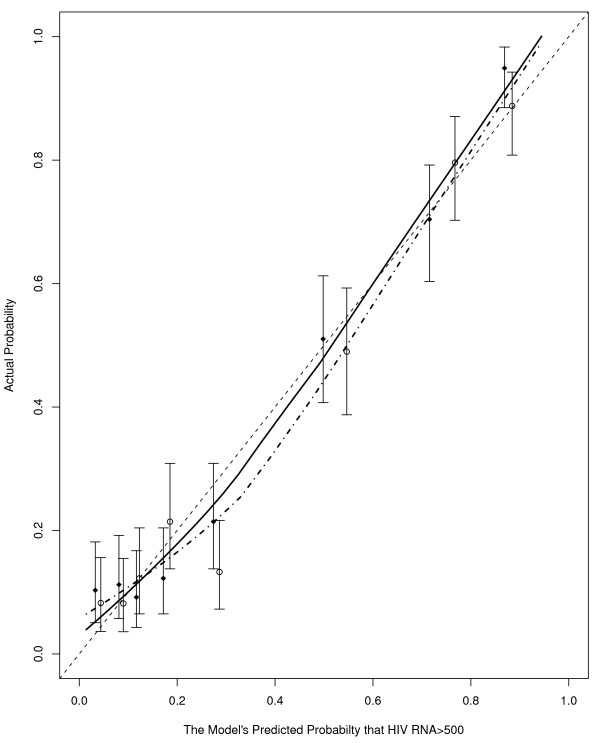
**Calibration curve**. A calibration curve resulting from the logistic model presented in Table 2, which shows good calibration overall when applied to the training (solid diamonds, solid line) and validation (open circles, dash-dot line) sets, despite that those with a predicted probability between 0.21 and 0.37 the actual probability appears to be lower than predicted in the validation set. Vertical lines correspond to 95% confidence intervals for the corresponding quintile group.

A logistic model was constructed based upon these final variables. The final logistic model is shown in Table 2 and shows approximately 20% decrease in odds having a HIV RNA above 500 copies/ml for every pg/cell higher of either MCH level or change in MCH (from pre-HAART levels). A calibration plot (Figure 1) demonstrated that the logistic model was fairly well calibrated as the curves lowess curves for the training (solid line) data set followed the 45 degree line. The area under the receiver operating curve (AUROC) was 0.85 (95% CI: 0.82-0.88). Using a predicted probability from the logistic model of >0.5 as having HIV RNA ≥500 copies/ml the sensitivity, specificity, positive and negative predictive values are shown in Table 3. The operating characteristics of this logistic model relative to the random-forest prediction approach resulted in a slight decrease in specificity (0.89 vs. random forest: 0.90) and an increase in sensitivity (0.70 vs. random forest: 0.67) with a positive predictive value of 0.77 and negative predictive value of 0.84 among the training set.

**Table 2:** Results of logistic model after screening for variables by the random forest approach***

	Beta Coefficient	Odds Ratio	Odds Ratio 95% Confidence Interval	p-value
Intercept	7.27	**		<0.0001
MCH (pg/cell)	-0.19	0.83	0.77, 0.89	<0.0001
Change in MCH (per pg/cell)*	-0.22	0.81	0.74, 0.88	<0.0001
CD4 (per 100 cells/mm^3^)	-0.32	0.73	0.65, 0.81	<0.0001
Change in Percent CD4 (per percent)*	-0.05	0.95	0.91, 0.99	0.008

* Change is relative to the pre-HAART value for an individual, thus a positive value for change in MCH indicates an increase in MCH for an individual from their pre-HAART value.

** The intercept has no OR interpretation.

*** To determine the predicted probability of having an HIV RNA > 500 copies/ml for an individual, take the value of each variable and multiply it by the corresponding Beta Coefficient. Take the sum of the resulting values and add the Intercept Beta Coefficient. This is the log(odds) that an individual has an HIV RNA value above 500 copies/ml. The predicted probability is then 1/(1+e(-log(odds)).

**Table 3:** Results of applying the logistic model to both the training set and validation set using a predicted probability of 0.5 as the cutoff*.

Training Set (N = 784)			
Model Classification*	HIV RNA > 500 copies/ml	HIV RNA ≤ 500 copies/ml	
HIV RNA > 500 copies/ml	192	57	PPV = 0.77
HIV RNA ≤ 500 copies/ml	83	452	NPV = 0.84
	Sensitivity 0.70 (95% CI: 0.64, 0.75)	Specificity 0.89 (95% CI: 0.86, 0.91)	

Validation Set (N = 784)			

Model Classification*	HIV RNA > 500 copies/ml	HIV RNA ≤ 500 copies/ml	
HIV RNA > 500 copies/ml	205	57	PPV = 0.78
HIV RNA ≤ 500 copies/ml	71	451	NPV = 0.86
	Sensitivity 0.74 (95% CI: 0.69, 0.79)	Specificity 0.89 (95% CI: 0.86, 0.91)	

* Individuals with probability >0.5 were classified as having HIV RNA > 500; Positive predictive value (PPV); Negative predictive value (NPV)

Change in MCHC from pre-HAART levels and MCHC were not included in the final model as these two variables were not significant in the logistic model (p = 0.71 and 0.36, respectively). Modeling a non-linear relationship of these two variables did provide a significant association (χ^2 ^= 18.64; 5 degrees of freedom; p = 0.002). However, when included in the model, these two variables and non-linear terms did not substantially improve prediction (AUROC = 0.86). Therefore, these variables were left out of the final logistic model in favor of a more parsimonious model.

**Figure 2:**
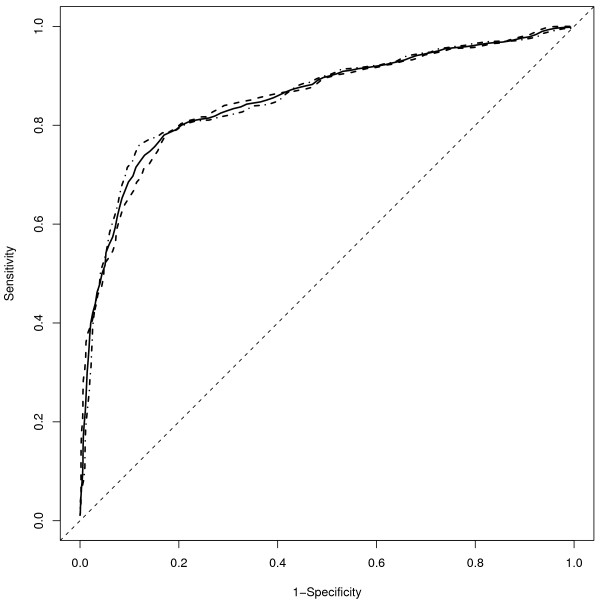
**Receiver operating characteristic curve**. The receiver operating characteristic curve (ROC) for the combined training and validation data set (solid line), training (dashed line), and validation (dash-dot line) data based upon the logistic model presented in Table 2.

The final random-forest applied to the validation set resulted in a sensitivity of 0.71 (95% CI: 0.66-0.76) and a specificity of 0.91 (95% CI: 0.88-0.93). These were not significantly different compared to the training set (sensitivity p = 0.27; specificity p = 0.67). When the logistic model was applied to the validation data set, the calibration curve (Figure 1, open circles and dashed-dot line) suggests that the actual probability was lower than the predicted. However, this was mainly for those with a predicted probability between 0.21 and 0.37 and otherwise the overall curve and confidence intervals suggest a fairly well calibrated model. Nevertheless, the sensitivity and specificity from the logistic model (Table 3), was not significantly different in the validation set as compared to the training set (p = 0.64 and p = 1.0, respectively). Furthermore, the AUROC in the validation set was unchanged at 0.85 (95% CI: 0.82-0.88) and the receiver operating curves for the training and validation data sets in addition to the two data sets combined were similar (Figure 2). Using the combined training and validation data sets, the point on the ROC curve that maximized both the sensitivity and specificity at both 0.80 was a cutoff in the predicted probability from the logistic model of 0.31.

For comparison to a logistic model based solely on CD4 at time of HIV RNA measurement, the training and validation data had an AUROC of 0.73 (95% CI: 0.70-0.77) and 0.75 (95% CI: 0.71-0.78), respectively indicating that CD4 by itself had a lower ability to discriminate between those who were and were not above 500 copies/ml. A cutoff in the predicted probability of 0.5 from this logistic model resulted in a sensitivity of 0.49 (95% CI: 0.44-0.55) and specificity of 0.87 (95% CI: 0.84-0.89) in the training data. Similar results were seen in the validation set (sensitivity: 0.55 [95% CI: 0.49-0.60]; specificity: 0.85 [95% CI: 0.82-0.88]). Inclusion of change in CD4 from pre-HAART levels slightly improved these results (AUROC of 0.77 and 0.78 for training and validation data sets, respectively).

## Discussion

There are two notable conclusions to this study. First, we expected traditional markers to be the most predictive (e.g. CD4, total lymphocyte counts) of current HIV RNA status. The importance of MCH and change in MCH was unexpected. There is a paucity of information on MCH with treatment and HIV RNA levels. Previous studies have suggested that mean corpuscular volume may change with NRTI use [13,14]. Another suggested that among treated individuals, those on an indinavir, nelfinavir, or saquinavir regimen had higher MCV and MCH than individuals on non-PI based regimens [15]. Perhaps the most compelling data is a recent study that examined hematological differences among Thai patients with and without antiretroviral therapy stratified by thalassemia (both alpha and beta) status [16]. Focusing on those without thalassemia, individuals treated with antiretrovirals had a higher MCH level (36.13 vs. 28.7 pg; p < 0.001) and higher MCV (107.26 vs 87.1 fL; p < 0.001) [16]. However, HIV RNA levels were not reported. Therefore, whether or not the importance of MCH is due to a correlation with HIV RNA levels or due to antiretrovirals, remains to be answered. While a significant portion of our study population was on a regimen containing a zidovudine, which has been associated with anemia [17-20], these antiretrovirals were not likely to have had an effect because treatment would have likely attenuated the association of MCH with an HIV RNA above 500 due to the inverse relationship. Furthermore, including variables indicating whether zidovudine was used did not significantly contribute to the random forest analysis. In the logistic model, the point estimates for MCH and change in MCH remained virtually unchanged (less than 5% of the estimate in Table 2) suggesting that zidovudine and stavudine are unlikely potential confounders of the MCH HIV RNA relationship. Furthermore, inclusion of zidovudine and stavudine in the model did not substantially improve the AUROC (0.86 vs. 0.85). Nevertheless, the relationship between HIV RNA and MCH should be further investigated in longitudinal studies to confirm this relationship.

Second, the results suggest that a binary rule for classifying individuals as either above or below 500 copies/ml is too simplistic. Rather it may need to be multiple markers as a set of complex binary partitions (random-forest) or a linear combination (on a logit scale) of multiple markers. The algorithmic random-forest approach has not been used extensively in HIV/AIDS applications but shows promise as a powerful tool to identify important variables that may classify individuals as above or below a certain HIV RNA threshold. As we have demonstrated, this approach may be used in conjunction with a regression model. For example, building a logistic model using a backwards stepwise selection approach with Akaike's information criteria upon our training data resulted in a model with 30 variables. Additionally it had an AUROC of 0.91 with a sensitivity of 0.75 and specificity of 0.92. However, this model was overly optimistic had an attenuated AUROC, sensitivity and specificity that was 0.84, 0.69, and 0.87, respectively. This demonstrates that our approach resulted in a much simpler model of 6 variables and an AUROC that remained constant at 0.85 in both the training and validation data sets. Thus our analysis did not result in an overly optimistic model (i.e. model was transportable to the validation set).

Our goal was to assess whether routinely collected clinical markers in addition to CD4 could potentially predict individuals who had an HIV RNA above 500 copies/ml after initiation of effective treatment. It is possible that additional information such as adherence data would improve the prediction and discrimination between those who do and do not have an HIV RNA above 500 copies/ml. Recent studies by Bisson [21] and Cambiano [22] have shown that adherence measures may be useful for detecting virologic failure and rebound, respectively. Thus inclusion of good adherence data is likely to improve our prediction model that focused on clinical markers.

We do not know whether these results will generalize to regions which need a method for identifying individuals whose HIV RNA levels remain above 500 copies/ml. Regional conditions may affect hematologic parameters such as MCH in ways (e.g., nutrition, endemic diseases, etc.) that would make the MCH less predictive. In addition, patients in a developed country can afford routine complete blood count testing, which may be less affordable and available in developing countries. Finally, the prevalence of HIV RNA suppression will also contribute to the usefulness of this predictor since the prevalence affects the positive and negative predictive values. However, our approach of utilizing a random forest to screen through variables to identify important predictors for a prediction model may be applied to resource limited settings.

We believe that our approach is more powerful for determining predictors of a suppressed viral load than previous approaches in that it was able to identify important prognostic markers from a large number of variables while providing a parsimonious model without loss (as compared to automatic backwards selection) in ability to discriminate between those who do and do not have an HIV RNA above 500 copies/ml. These methods could be used to determine whether the MCH or other biomarkers can be used in resource-limited settings where the viral load is not routinely available.

## Competing interests

The authors declare that they have no competing interests.

## Authors' contributions

BL contributed to the design and analysis of the data and drafted the manuscript. GC contributed to the interpretation of the data and revising the manuscript. SJG contributed to the design and interpretation of the data and manuscript revisions. RDM contributed to the acquisition and interpretation of the data and manuscript revisions. All authors have given final approval of the manuscript.
